# Effect of Pyrolysis on iron-metal organic frameworks (MOFs) to Fe_3_C @ Fe_5_C_2_ for diesel production in Fischer-Tropsch Synthesis

**DOI:** 10.3389/fchem.2023.1150565

**Published:** 2023-04-11

**Authors:** Saleem Munir, Muhammad Amin, Naseem Iqbal, Amjad Iqbal, Ayman A. Ghfar

**Affiliations:** ^1^ U.S.-Pakistan Centre for Advanced Studies in Energy (USPCAS-E), Department of Energy Systems Engineering, National University of Sciences and Technology, Islamabad, Pakistan; ^2^ Departament d’Enginyeria Química (DEQ), Universitat Rovira i Virgili (URV), Tarragona, Spain; ^3^ Department of Energy Systems Engineering, Seoul National University, Seoul, South Korea; ^4^ Department of Materials Technologies, Faculty of Materials Engineering, Silesian University of Technology, Gliwice, Poland; ^5^ Department of Mechanical Engineering, CEMMPRE-Centre for Mechanical Engineering, Materials and Processes, University of Coimbra, Coimbra, Portugal; ^6^ Department of Chemistry, College of Science, King Saud University, Riyadh, Saudi Arabia

**Keywords:** syngas, effect of pyrolysis, diesel, air pollution reduction, MOF catalyst, fuel

## Abstract

The Fischer-Tropsch Synthesis (FTS) is a significant catalytic chemical reaction that produces ultra-clean fuels or chemicals with added value from a syngas mixture of CO and H_2_ obtained from biomass, coal, or natural gas. The presence of sulfur is not considered good for producing liquid fuels for(FTS). In this study, we reveal that the presence of sulfur in ferric sulfate Fe_2_(SO_4_)_3_ MOF provides the high amount, 52.50% of light hydrocarbons in the carbon chain distribution. The calcined ferric nitrate Fe(NO₃)₃ MOF reveals the highest 93.27% diesel production. Calcination is regarded as an essential factor in enhancing liquid fuel production. Here, we probed the calcination effect of Metal Organic Framework (MOF) on downstream application syngas to liquid fuels. The XRD results of MOF. N and P. MOF.N shows the formation of the active phase of iron carbide (Fe_5_C_2_), considered the most active phase of FTS. The scanning electron microscopy (SEM) images of iron sulfate MOF catalyst (P.MOF.S) reveals that the existence of sulfur creates pores inside the particles due to the reaction of free water molecules with the sulfur derivate. The surface functional groups of prepared MOFs and tested MOFS were analyzed by Fourier transforms infrared spectroscopy (FT-IR). The thermal stability of prepared MOFS was analyzed by Thermo gravimetric analysis (TGA). The surface areas and structural properties of the catalysts were measured by N2-Physiosorption technique.

## Graphical Abstract

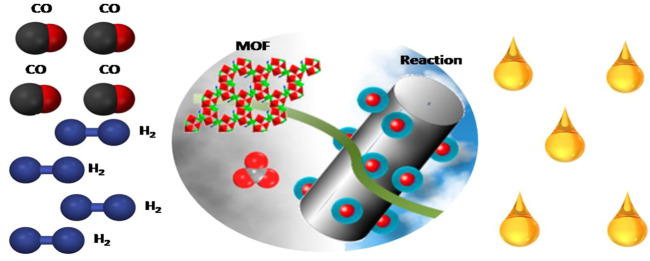

## 1 Introduction

Depleting the fossil fuels the scientific community is looking for alternate raw materials to meet human needs as a result of climate changes brought on by anthropogenic emissions and fossil fuel feedstock (Capellán-Pérez et al., 2014). Biomass is the second main source of organic carbon on our planet (Ahorsu et al., 2018) has been identified as a source of resources that is renewable and possibly sustainable (Esposito and Antonietti, 2015)-(amin et al., 2022). Therefore, it is not surprising that several biomass conversion techniques have been created in recent years (Corma Canos et al., 2007). Utilizing this approach frequently could also help with reducing greenhouse gas emissions and addressing other issues (Höök and Tang, 2013)-(Ur et al., 2022). In addition, renewable energy sources might not always be a good replacement for fossil fuels in some circumstances (Amin et al., 2022a). This might be the case for fuels used in transportation, when biomass could instead offer worthwhile substitutes, such as fuels derived from biomass (or befouls). Due to the fact that these fuels are composed of liquid hydrocarbons, the mixtures must contain alkenes with certain carbon chain ranges in order to comply with the aforementioned requirements (West et al., 2008)-(Amin et al., 2022b).

Creating diesel and jet fuels from biomass involves a number of C-C bond-forming techniques (Wang and Tao, 2016), including the oligomerization of bioethanol and the Fischer-Tropsch reaction of syngas produced from biomass (Gruber et al., 2021). The coordination of inorganic nodes and multidentate organic linkers, typically carboxylic acids and their derivatives, results in MOFs, which are three-dimensional, porous materials. Recently, Metal-organic frameworks (MOFs) constructed from inorganic nodes and organic linkers have gained huge attention for Fischer Tropsch synthesis due to their diversity in structural properties (Cho et al., 2020). Due to their unique structure, textural properties, and atomic metal dispersion competencies, MOFs are considered promising precursors for synthesizing catalysts for Fischer Tropsch synthesis (FTS) (Santos et al., 2015). Hematite (Fe_2_O_3_), magnetite (Fe_3_O_4_), and siderite (FeCO_3_) are the three main ores of iron (Fe), which is one of the metals with the greatest abundance in the earth’s crust. Iron nanoparticles with distinctive structures are produced by MOFs-mediated Fe-based catalysts (MeharNisa et al., 2020). Iron-based MOFs have been intensively investigated for higher activity from water-gas shift reactions due to the simultaneous formation of various iron phases such as α-Fe, Fe_3_O_4_, Fe_4_C, θ-Fe_3_C, χ-Fe_5_C_2_, Fe_7_C_3_, and ε′-Fe_2.2_C during the FTS reaction. The iron carbide (χ-Fe_5_C_2_) has been well-reported as one of the most active iron phases for better selectivity in FTS reactions (Cho et al., 2020).

The stability of MOFs is influenced by various factors, including the coordination bond strength between inorganic nodes and organic ligands, the configuration of organic ligands, species of metal ions, hydrophobicity of pore surfaces, and their pyrolysis temperature. Pyrolysis of MOFs has been used to convert the MOFs into porous metal nanoparticles to enhance the performance of activity and stability of the catalyst (An et al., 2016). The pyrolysis temperature conditions and time duration has been reported as one of the most important parameters to stable the structural properties of the iron carbide χ-Fe_5_C_2_ in the catalyst (Cho et al., 2020). In earlier research, ferric sulfate was employed in the MOF and only 5%–8% light hydrocarbons were obtained. But in our study, we used pyrolysis ferric sulfate MOF (MOF.S) in the Fischer Tropsch synthesis, and we were able to obtain 52.50% of light hydrocarbons in the C1 to C4 range. The annealing at high-temperature tenability the pores of MOFs to enhance the catalytic product selectivity. High-temperature pyrolysis achieved more rapid contact between Fe and C to promote iron carbide formation during the catalyst preparation. Moreover, the formation of high metals such as Fe particles leads to the high carbide phase resulting in an exceptionally high activity and good stability of FTS reaction. Therefore, in this study, pyrolysis all used MOF was annealed at a temperature of 600°C (rate 5 °C/min) for 3 h (Otun et al., 2020).

According to our knowledge, there is a lack of investigation into the production of diesel through the pyrolysis of ferric nitrate (P.MOF.N) and pyrolysis of ferric sulfate (P.MOF.S) metal organic framework. By using MOF, we revealed that ferric nitrate and ferric sulfate have significant impacts on the production of diesel. Hence, in this article, we investigate the two different Fe-MOF syntheses from iron precursor ferric nitrate [Fe (NO_3_)_3_] and ferric sulfate [Fe_2_ (SO_4_)_3_] pyrolysis at high temperature (600°C) and investigate their effect on hydrocarbon production for Fischer Tropsch synthesis. The obtained MOFs from ferric nitrate and ferric sulfate were classified in this article as MOF. N and MOF. S, respectively. These two iron precursors are selected due to their low cost and good selectivity for Fischer-Tropsch synthesis reactions. The high-temperature pyrolysis conditions eliminated the existence of nitrogen, sulfur, and chlorine from the MOF and gave birth to an utterly iron carbide phase that is worthy for the production of C^12+^ hydrocarbons was investigated.

## 2 Experimental section

### 2.1 Catalyst preparation

**FIGURE 1:**
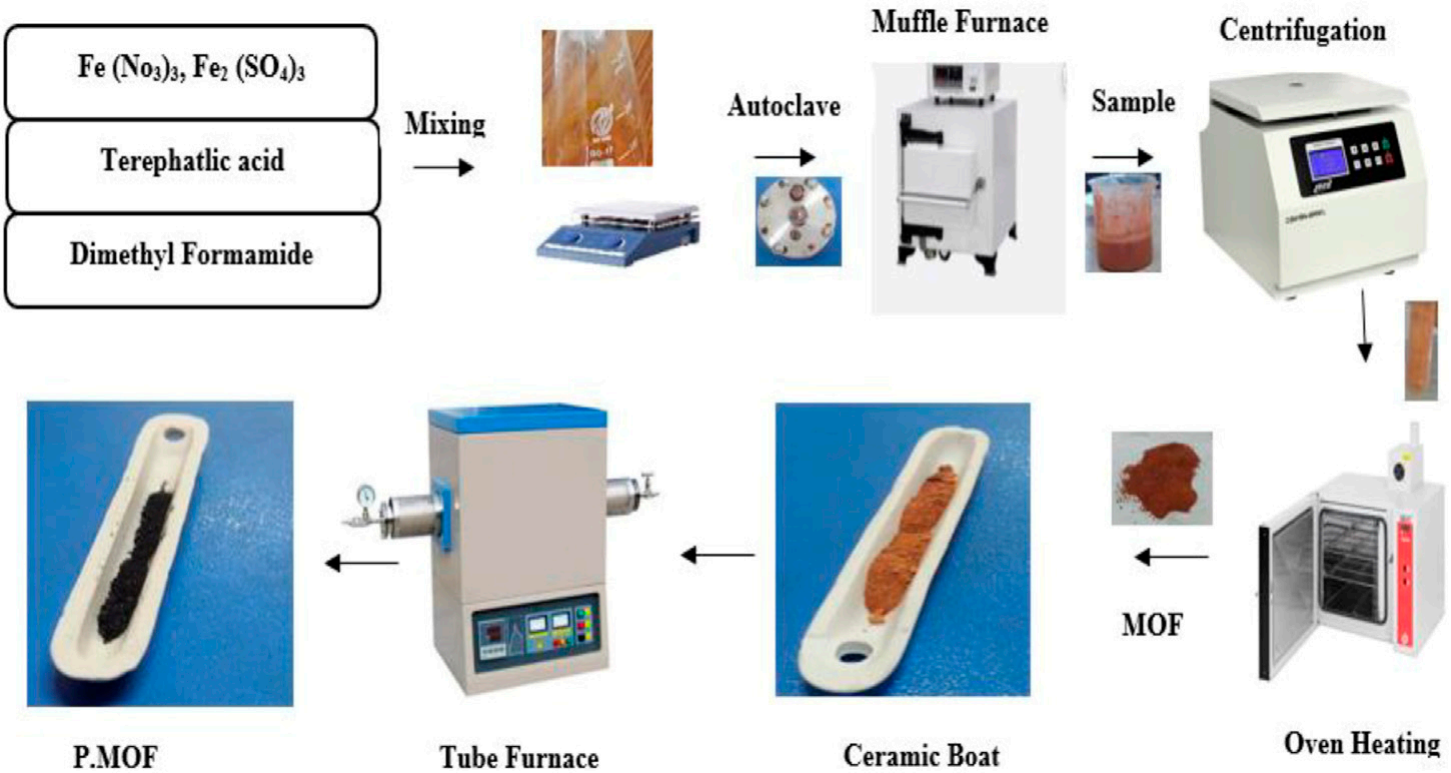
Preparation of MOFs and Pyrolysis MOFs by solvothermal method.

The solvothermal method was used to prepare the MOF catalysts (Gruber et al., 2021). Ferric nitrate [Fe (NO_3_)_3_] (8.1 g) and Terephatlic Acid [C_8_H_6_O_4_] (8.3 g) we remixed in 250 mL Dimethyl Formamide [C_3_H_7_NO] and set for stirring until homogenous solution achieved. The mixed solution was put in the autoclave, and then the reaction was carried out in a muffle furnace at a temperature of 150°C for 2 h (Cho et al., 2020). After cooling to room temperature, the sample was centrifuged at 6,000 rpm for 5 min. The powder particles were kept in the oven for drying at 80°C for 24 h. Finally, using an inert nitrogen environment, the MOF was annealed in a tube furnace. (Santos et al., 2015). in order to create composite systems of Fe3O4 and carbon. The sample was put into a ceramic boat, kept inside the tube furnace, and started a temperature that is gradually heated (5°C/min) to 600°C for 3 h. The same procedure was applied to prepare catalysts through ferric sulfate [Fe_2_(SO_4_)_3_]. The sample was cooled at room temperature and then kept all samples in the sealed sample bottle. Figure 1 explains the steps involved in the preparation of MOF and pyrolysis MOF catalyst. Various characteristic analysis methods were examined. to determine the properties of prepared MOF and pyrolysis MOF catalysts. Utilizing a scanning electronic microscope and energy-dispersive X-ray analysis, changes in surface morphology were examined. (JEOL JSM 6490 LA) (MeharNisa et al., 2020). X-ray diffraction determined crystal structure (XRD-D8 advanced by Bruker Germany) determined crystal structure. The thermal degradation behavior was observed with the TGA analysis (Model: DTG-60 H). The functional group is detected from the FT-IR spectrum technique (xyz).

### 2.2 Catalytic performance test

**FIGURE 2:**
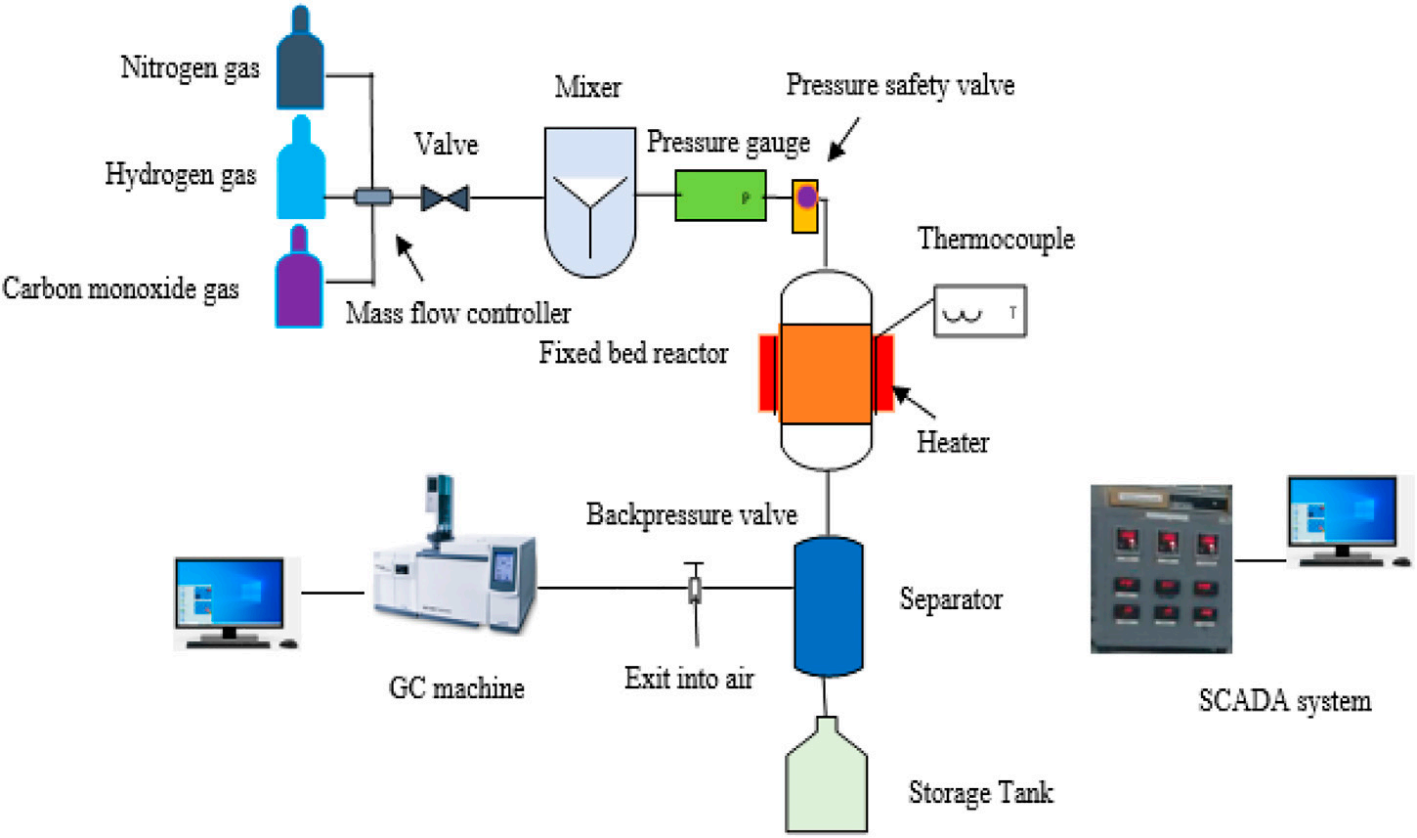
Schematic diagram of the fischer tropsch synthesis setup.

A stainless steel fixed bed reactor with a 10 cm length and 3.3 cm diameter was used to test the catalytic performance of FTS. A reactor was loaded with one gram of ready MOF and pyrolysis MOF catalyst. The reactor was a fixed bed reactor to reduce the catalyst for 5 hs at 450°C with a flow of 30 sccm hydrogen gas and nitrogen flow gas (10°sccm). The reactor was then cooled to 200°C in order to facilitate the reaction at 20 pressures and a 1:1 CO/H_2_ ratio. In order to regulate the pressure, a back-pressure regulator was utilized. A sample of the liquid product was taken from each of the two separators. The tail gases were examined online by gas chromatography (Shimadzu GC-2010 Plus). Gas chromatography-mass spectrometry (GCMS-QP2020) analysis was used to determine a liquid sample collected from a separator was analyzed for composition. 120°C was chosen as the injection temperature. The column flow rate was set at 1.44 mL/min, and helium was employed as the carrier gas at a pressure of 89.3 KPa. 30 min passed during the entire operation, and the total flow was 158.9 mL/min. To determine the product selectivity, the peak area in the GCMS findings is employed. The FTS plant used for this experiment is shown schematically in Figure 2 (Amin et al., 2022c).

## 3 Results and discussions

**FIGURE 3:**
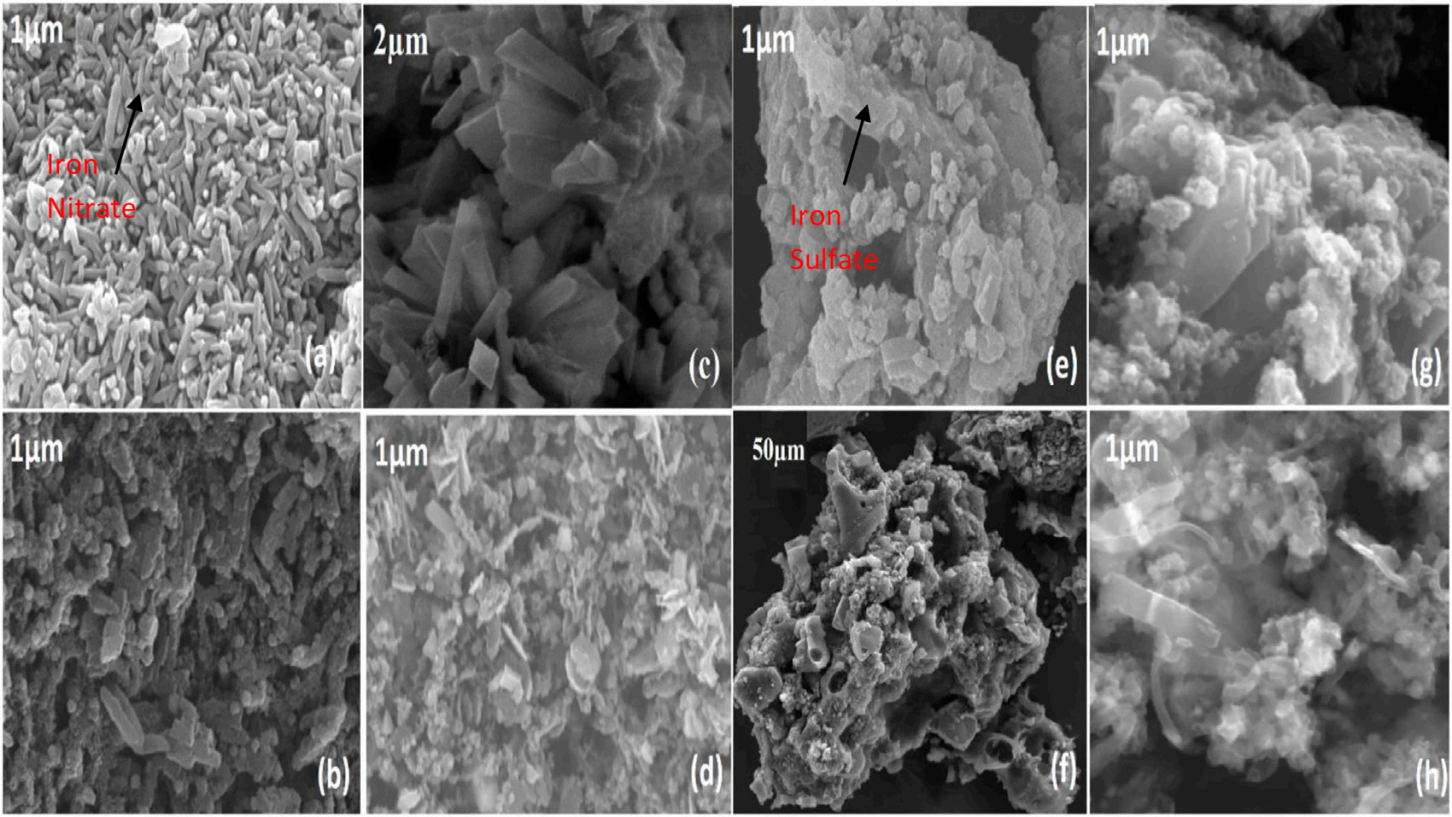
Scanning electron microscopy (SEM) of ferric nitrate (MOF.N) in **(A)** and ferric sulphate (MOF.S) MOF in **(E)**, pyrolysis (P.MOF.N) and (P.MOF.S) catalysts.

The scanning electron microscopy (SEM) images of prepared MOF and pyrolysis MOF are shown in Figure 3; Figure 3A reveals that the iron nitrate MOF. N particles are needle-like structures, and entire iron particles were distributed with uniform formation. Figure 3B shows that these iron particles shape is deformed due to high temperatures calcination. The SEM images of MOF. N (c) demonstrates that the MOF. N iron particles maintain their original needle structure but attach with the carbide particles to form the iron carbide formation. The formation of iron carbide (Fe_5_C_2_) enhanced the carbon chain in Fischer tropsch synthesis (Lyu et al., 2020). Figure 3D describes that the after reaction in fixed bed reactor the pyrolysis iron nitrate metal organic framework (P.MOF.N) particles made a much more stronger bond with the carbon particles to form the formation of iron carbide (Fe_5_C_2_). The formation of iron carbide phase in MOF. N was observed less as compared to pyrolysis P. MOF.N. (Amin et al., 2022c), (Mahmood et al., 2022)

Figure 3E shows that the MOF prepared from iron sulfate (MOF.S) shows the presence of sulfur particles attached to the iron and carbon particles. After the calcination under the tube furnace atmosphere, the (f) presence of sulfur particles is reduced, but pores are formed inside the particles. This pore formation is due to the sulfur reacting with the free water molecules to generate heat. Figure 3G reveals that the after reaction in fixed bed reactor the P. MOF.S the iron particles with sulfur still there that lower the performance of the FTS reaction (Kritzinger, 2002). However, Figure 3H shows that after the FTS reaction, the pyrolysis MOF. S completely deforms its shape.

The surface areas and structural properties of the catalysts were measured by the Brunauer–Emmett–Teller (BET) method (Micromeritics ASAP 2010 instrument, United States). The pore size distribution and pore volume therein were evaluated by the Barret–Joyner–Halenda (BJH) method. All the catalyst samples were degassed at 200°C for 10 h before N_2_physisorption.

The corresponding pore size distributions in Table 1 demonstrate that the pore sizes range within the mesoporous range (2–50 nm). Here, the pore size distribution was calculated using the adsorption branch. The pyrolyzed carbon materials in this work had more regular pore structures than activated carbon, which has a three-dimensional nanostructure with micropores, mesopores, and macropores. This conclusion is supported by the comparatively narrow pore size distribution in the mesoporous range. According to the BET method, the specific surface areas of the catalysts for pyrolysis ferric nitrate (P.MOF.N) and pyrolysis ferric sulphate (P.MOF.S) are 238 and 240 m2 g1 respectively. This is because after the calcinations, the surface area increased, demonstrating the porous structure of the catalyst. More adsorption sites are made possible by a high surface area for the reactant.

**TABLE 1:** N2- Physiosorption analysis of MOFs catalysts.

Catalysts	Surface area (m^2^ g^-1^)	Pore volume (cm^3^ g^-1^)	Pore size (nm)
Ferric nitrate (MOF.N)	186	0.27	3.07
Ferric sulphate (MOF.S)	202	0.32	3.42
Pyrolysis ferric nitrate (P.MOF.N)	238	0.35	3.41
Pyrolysis ferric sulphate (P.MOF.S)	240	0.30	3.42

**FIGURE 4:**
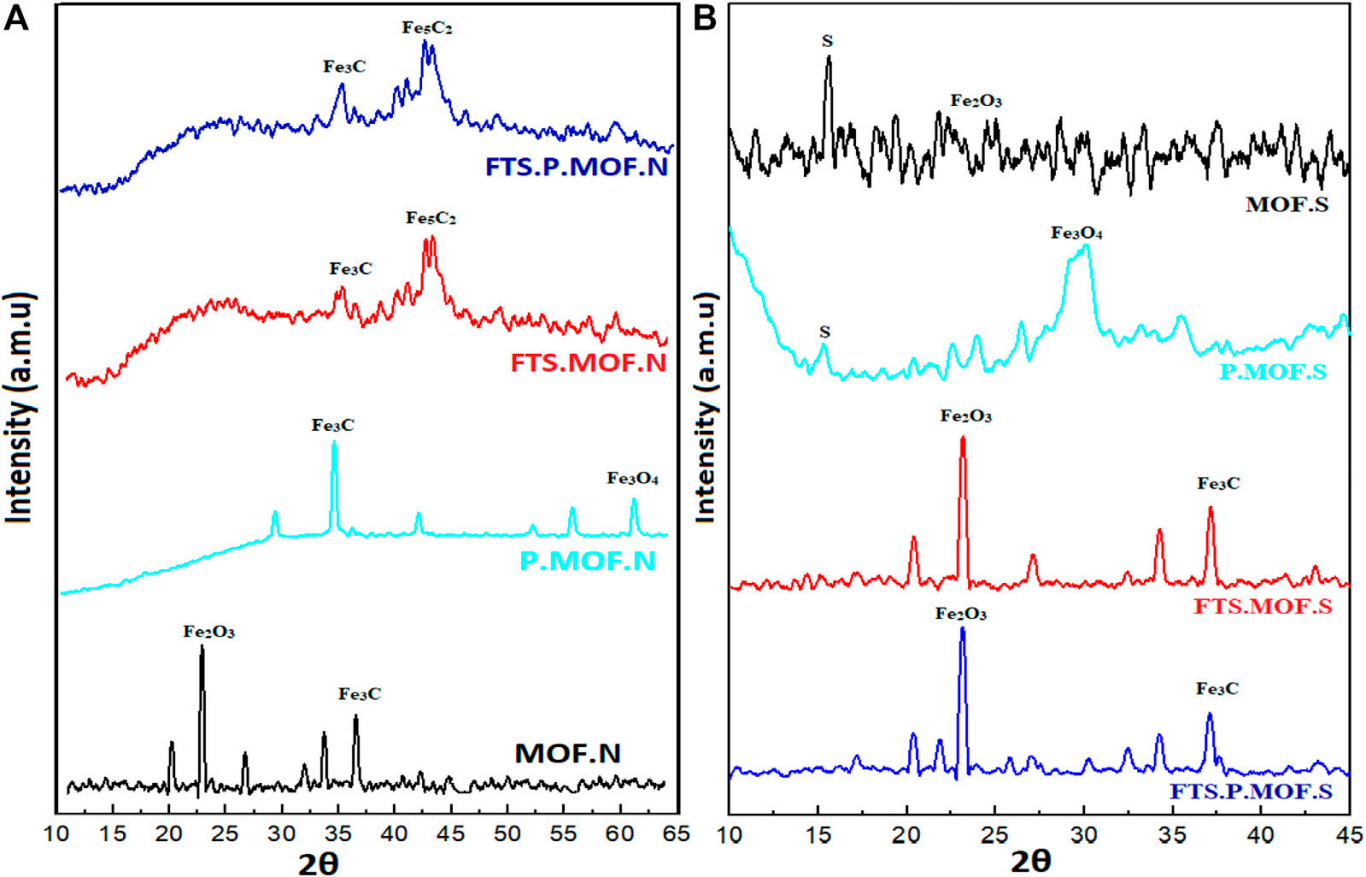
XRD analysis of **(A)** ferric nitrate and **(B)** ferric sulphate prepared MOF, pyrolysis MOFs and FTS MOFs.

The XRD results of MOFs and pyrolysis MOFs are shown in Figure 4A XRD peak of MOF. N at 24° (Xiong et al., 2015) indicates the formation of hematite (Fe_2_O_3_) with PDF # 33–0664, and at 36°, the formation of iron carbide (Fe_3_C) (Yang ZhangLiuNingHanLiu et al., 2019) with PDF # 00–003–1056 occurs during the preparation. As the MOF is calcined in a tube furnace under the inert nitrogen gas atmosphere, the peak at 29° (Chun et al., 2014) shows the presence of magnetite (Fe_3_O_4_) with PDF # 19–0770, and the peak at 34.8° indicates the presence of iron carbide (Fe_3_C). After the FTS reaction of MOF. N and P. MOF.N in a fixed bed reactor, highly active phase iron carbide (Fe_5_C_2_) with PDF # PDF#06–0696 formation occurs at an angle 44°.Moreover, the peak at 45 indicates the presence Iron sulfide with PDF#65–2567. This active phase leads the production of C_12_-C_22_ (diesel) in the product chain (Lyu et al., 2020).

Figure 4B demonstrates the XRD peaks of MOF prepared by ferric sulfate. The MOF. S XRD peak at angle 16 reveals the existence of sulfur. A large number of stack layers in MOF. S are due to the derivate of sulfur. Sulfur is not considered good in Fischer tropsch synthesis because it creates choking during the reaction, reducing the reaction’s performance. Even after the calcination in the tube furnace, the P. MOF.S very small sulfur peak was observed. The P. MOF.S XRD peak at angle 29° shows the formation of magnetite (Fe_3_O_4_). After the FTS reaction, both the used MOFs reveal the presence of hematite (Fe_2_O_3_) at an angle of 24° and iron carbide (Fe_3_C) at 36.8° (Awwad et al., 2015). The formation of hematite during the FTS reactions leads to the production of light hydrocarbons, as observed in GCMS results.

**FIGURE 5:**
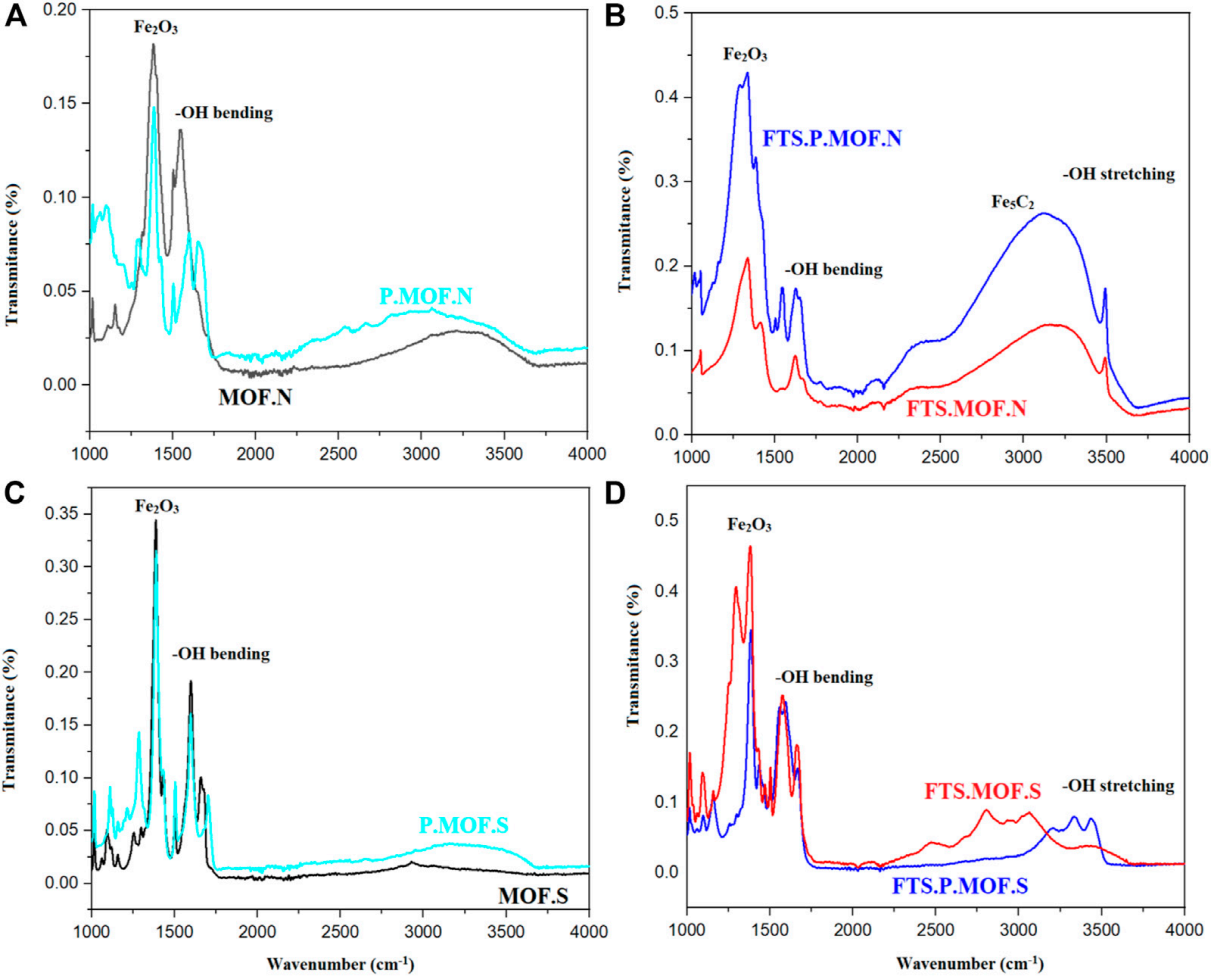
FTIR analysis of **(A, B)** ferric nitrate and **(C, D)** ferric sulfate prepared MOF, pyrolysis MOFs and FTS MOFs.

Figure 5 shows the surface functional groups of prepared MOFs, and tested MOFS were analyzed by Fourier transform infrared spectroscopy (FT-IR). The peaks at the wave number of 1331–1365 cm^-1^ indicate the bond formation of Fe_2_O_3_ (xi Song et al., 2021). The peaks at the wave number of 1600–1632 cm^-1^ indicate the–OH bending vibrations. This OH bending vibration indicates abundant functional groups in the surface layer (Ma et al., 2020). The interpretation of the IR spectra can be used to gain insights into the role of surface -OH groups in catalysis. For example, the presence of -OH groups on the surface of a MOF can enhance its catalytic activity by providing Lewis acid or base sites for adsorption and reaction with substrates. In addition, the presence of -OH groups can also influence the stability and reactivity of the catalyst by affecting its electronic structure and surface acidity.

Furthermore, the type and location of -OH groups on the surface of MOFs can also affect their catalytic properties. For instance, -OH groups located near the metal center can act as coordinatively unsaturated sites, facilitating reactions involving metal coordination. Meanwhile, -OH groups located on the organic linkers can act as hydrogen bond donors or acceptors, enabling selective adsorption and reaction with certain substrates.

The MOF prepared by ferric nitrate indicates the active bond phase of iron carbide (Fe_5_C_2_) at a wave number of 2848–2917 cm^-1^ [545]. Figures 5B, D shows the hydroxyl groups causes a stretching vibration. (Nalbandian et al., 2015).

**FIGURE 6:**
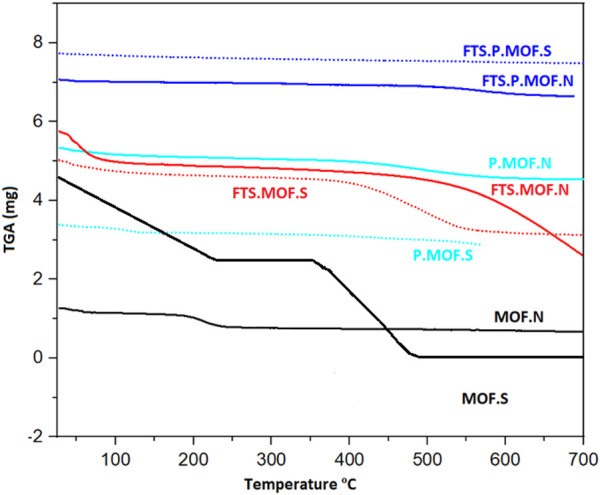
TGA analysis of ferric nitrate and ferric sulfate prepared MOF, pyrolysis MOFs and FTS MOFs.

The results are shown in Figure 6. Free molecules of water that were present in the interlayer gap were evaporated, which was responsible for the removal of physically adsorbed water from the ready samples in a temperature range of 110–250°C. This was carried out to assess the thermal stability of MOF, P. MOF, and FTS run catalysts carried out under a N_2_ flow. The decomposition of goethite first takes place at temperatures between 200°C and 250°C (Eq. (1)) (Shakeri et al., 2021) (Einemann M, Neumann F, Thomé A.G, Wabo S G, Roessner F, 2020)- (Shakeri J, Joshaghani M, Hadadzadeh H, Shaterzadeh M.J, 2021). FeO is formed after Fe_2_O_3_ (hematite) decreases. Iron oxide and CO are reacted at 250°C–300°C to produce Fe_2_O_3_.

Iron oxides are reduced to produce iron carbide carbonaceous material related to the generation of CO_2_ at higher temperatures above 350°C, as shown by equations. (8-10) (Pérez et al., 2019) (Pérez S, Mondragón F, and Moreno A, 2019). The formation of iron carbide is also confirmed in XRD results.

The CO conversion is an important factor that can influence the product selectivity in CO oxidation reactions. The CO conversion is defined as the percentage of CO that is converted to CO2, while the product selectivity is the percentage of the converted CO that forms a specific product (e.g., CO2).

In general, increasing the CO conversion can lead to a higher overall product yield, but it may also lead to the formation of undesired byproducts, which can decrease the selectivity towards the desired product. Therefore, it is important to optimize both the CO conversion and product selectivity to achieve the desired overall reaction performance.

$$2\;FeO\;{\left(OH\right)}_{\left(s\right)}\to {Fe}_{2}O_{3\left(s\right)}+{H_{2}O}_{\left(g\right)}$$ (1)

$$3\;{FeCO}_{3\left(s\right)}\;\to \;{Fe}_{3}O_{4\left(s\right)}+2{CO}_{2\;\left(g\right)}+{CO}_{\left(g\right)}$$ (2)

$${FeCO}_{3\left(s\right)}\;\to \;{FeO}_{\left(s\right)}+{CO}_{2\left(g\right)}$$ (3)

$${FeC}_{2}O_{4•}2H_{2}O_{\left(s\right)}\;\to \;{FeC}_{2}O_{4\left(s\right)}+2H_{2}O_{\left(g\right)}$$ (4)

$$3\;{FeC}_{2}O4_{\left(s\right)}\;\to \;{Fe}_{3}O_{4\left(s\right)}+4{CO}_{\left(g\right)}+2{CO}_{2\left(g\right)}$$ (5)

$$3\;{Fe}_{2}O_{3\left(s\right)}+{CO}_{\left(g\right)}\;\to \;2\;{Fe}_{3}O_{4\left(s\right)}+{CO}_{2\left(g\right)}$$ (6)

$$6\;FeO\;{\left(OH\right)}_{\left(s\right)}+{CO}_{\left(g\right)}\;\to \;2\;{Fe}_{3}O_{4\left(s\right)}+{CO}_{2\left(g\right)}+3H_{2}O_{\left(g\right)}$$ (7)

$${FeCO}_{3\left(s\right)}\to FeO+{CO}_{2\left(g\right)}$$ (8)

$$x\;{Fe}_{3}O_{4\left(s\right)}+x\;{CO}_{\left(g\right)}\to 3\;{Fe}_{x}\;C\left(s\right)+x{CO}_{2\left(g\right)}$$ (9)

$$x\;{FeO}_{\left(s\right)}+x\;{CO}_{\left(g\right)}\;\to \;{Fe}_{x}C\;\left(s\right)+{xCO}_{2\left(g\right)}$$ (10)

The presence of sulfur might create a possible reaction during the pyrolysis and the FTS reaction (Eddy et al., 2022). Sulfur and oxygen react to produce sulfur dioxide. Further, the sulfur dioxide reacts with the oxygen to form sulfur trioxide. When the free water molecules react with sulfur trioxide, it forms sulfuric acid. The reaction between water and sulfuric acid generated the heat that creates the formation of the pores that is observed in SEM images of P. MOF. During the FTS reaction, the elemental sulfur reacts with iron to form the iron sulfide. Iron sulfide reacts with oxygen and includes the (Fe_2_O_3_) observed in the XRD peaks (Warneck, 2018).

Fe+S→ FeS (11)

$$4FeS+7O_{2}\to 2{Fe}_{2}O_{3}+4{SO}_{2}$$ (12)

### 3.1 Carbon chain distribution

**FIGURE 7:**
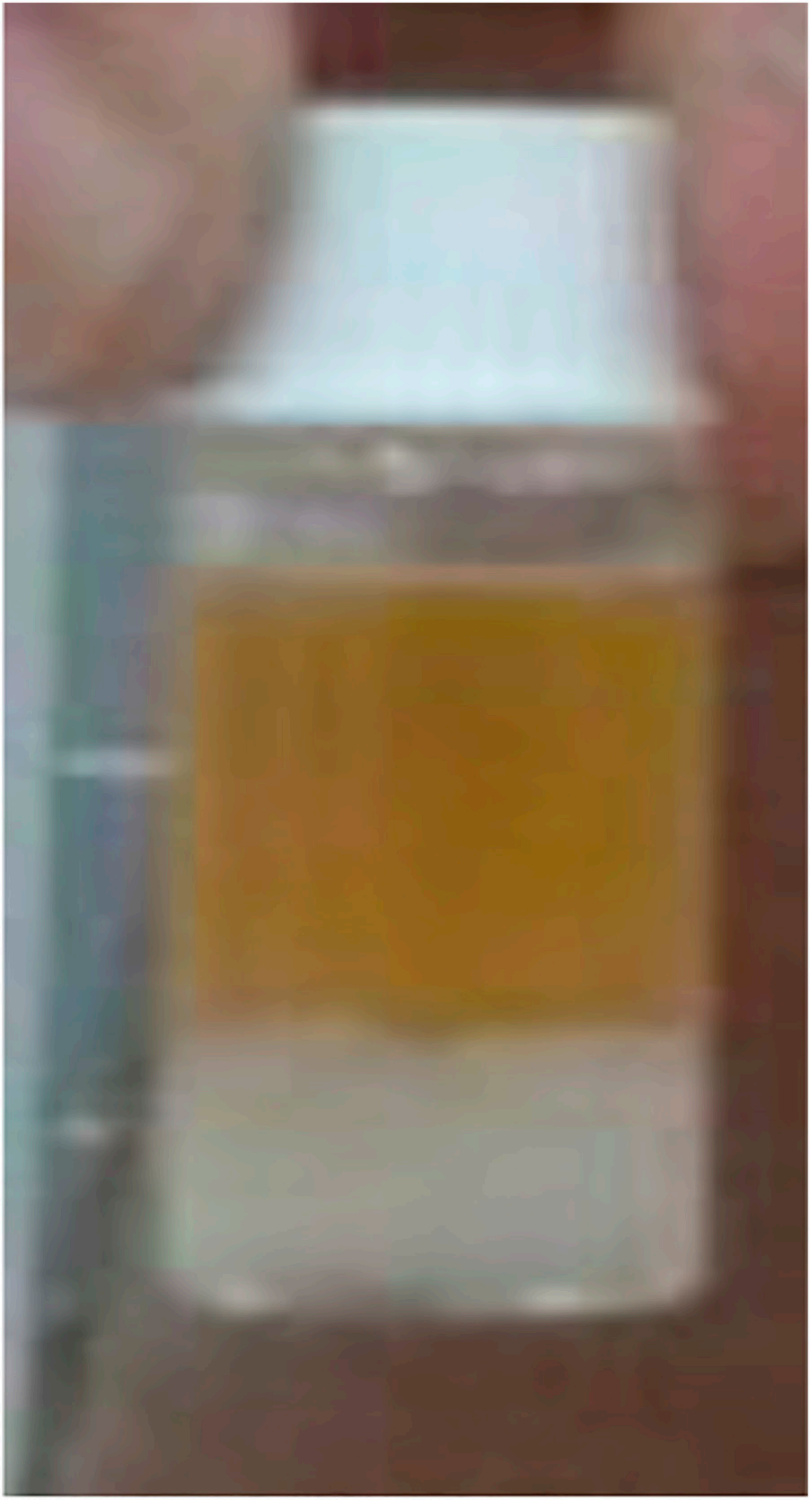
FT-product collection.

A valuable transportation fuel can be produced using the FTs process, which can also create the hydrocarbon chains C5-C11 (for gasoline) and C12-C22 (for diesel). MOF and pyrolysis analyzed the performance of FTS catalysts MOF prepared from ferric nitrate and ferric sulfate. At 150°C, CO dissociation occurred during reduction, and the most active phase of the reaction that resulted from the reaction of the ferric nitrate-based catalyst was thought to be the synthesis of Fe_5_C_2_. (Cho et al., 2020). However, the GCMS results were different in the case of the ferric sulfate catalyst; it exhibited the presence of sulfur to generate the highest amount of light hydrocarbons. The sulfur decreases the activity of the catalyst that not leads to the formation of the iron carbide phase (Bambal et al., 2014). To prevent evaporation, the sample wassealed into glass vials. Figure 7: After being removed from separators following thereaction. Two distinct layers can be seen in the samples that were collected; the upper brown layer is made up of C5+ hydrocarbons, while the lower white layer is oxygenated. Figure 6 of the results, which depict all potential product chemicals, reveals various peaks. The percentages of each hydrocarbon in the combination are listed in total in Table 2.

**TABLE 2:** Carbon chain distribution with oxygenates and non-oxygenates.

Catalysts	Temperature (^o^C)	Distribution of carbon chain (%)				Oxygenates (%)	Non-oxygenates (%)
		C_1_-C_4_	C_5_-C_11_	C_12_-C_22_	>C_23_		
		Light hydrocarbons	Gasoline	Diesel	Lubricating oil		
MOF.N	200	0.02	4.69	85.40	9.79	30.5	69.5
P.MOF.N	200	––	1.86	93.27	4.87	11.5	88.5
MOF.S	200	52.50	14.45	28.02	4.93	14.6	85.4
P.MOF.S	200	––	1.04	85.42	13.54	17.5	82.5

**FIGURE 8:**
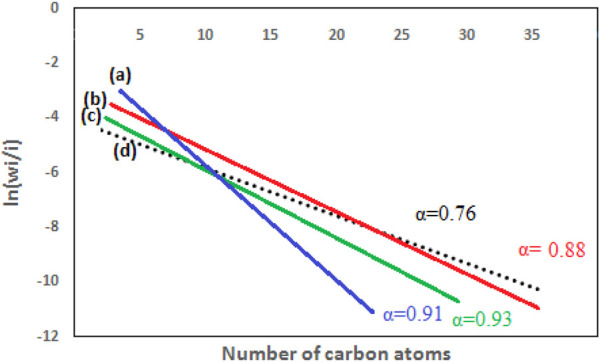
Product composition: **(A)** P. MOF.S **(B)** MOF. N **(C)** P. MOF.N and **(D)** MOF.S.

MOFs prepared by ferric nitrate (P.MOF.N) and ferric sulfate (P.MOF.S) revels the existence of light hydrocarbons. Due to the presence of sulfur in ferric sulfate MOF, the light hydrocarbon amount was observed as high at 52.50%. The derivate of sulfur could not enhance the carbide formation during the FTS reaction. In this study, the MOF.N contributed to the diesel production by 85.40%, but this diesel Production increased by 93.27% (Figure 8) when the calcined P.MOF.N was used in FTS reactions. The same pattern was observed as the MOF prepared by ferric sulfate (MOF.S) reveals just 28.02% diesel production. However, calcined P. MOF.S dramatically increased diesel production by 85.42%. This study demonstrates that, first; the presence of sulfur was the leading cause of lower diesel production. Second, calcination has a significant impact on the higher carbon chain distribution.

Diesel is generated as the main component, with an approximate value of 0.9 in LTFT (low temperature fischer tropsch), according to Anderson-Schulz-Flory (ASF) Distribution. Under HTFT (high temperature fischer tropsch) situations, gasoline needs slightly lower levels of 0.7–0.8. (Claeys and Van Steen, 2004).

$$\propto =\left(0.2332.\left(\frac{yCo}{yCo+{yH}_{2}}\right)+0.633\right).\left(-0.0039.\left(\left(T\left({}^{\circ}C\right)+273\right)-573\right)\right)$$ (13)

$$\frac{Mn}{n}={\left(1-\alpha \right)}^{2}.\alpha^{\left(n-1\right)}$$ (14)

$$\ln\alpha =nln\alpha +\ln\left[\frac{{\left(1-\alpha \right)}^{2}}{\alpha }\right]$$ (15)

The ASF model is illustrated in Eq. 13 to determine the “α”. The “α” is determined using the gradient of the linearized expression in the log Mn/n against the n plot, which is given as Eqs 14, 15 (Aluha et al., 2017)- (Martin, 2016). Figure 6 shows the computed value “α” using the ASF model.

Some of the recent studies about the Iron MOF catalyst demonstrate that the ferric nitrate and ferric sulfate have low production of diesel range fuel. However, in our studies the after the pyrolysis of ferric sulfate metal organic frameworks (P. MOF. S) and pyrolysis of ferric nitrate metal organic framework (P.MOF.N) catalyst have been considered the diesel range fuel at low temperature around 200°C in Fischer Tropsch synthesis reaction.

## 4 Conclusion

Iron-based MOFs have been intensively investigated for higher activity from water-gas shift reactions due to the simultaneous formation of various iron phases such as α-Fe, Fe_3_O_4_, ε′-Fe_2.2_C,θ-Fe_3_C, χ-Fe_5_C_2_,, and during the FTS reaction. High-temperature pyrolysis achieved a more rapid contact between Fe and C to promote iron carbide formation during the catalyst preparation. The prepared calcined MOFs exhibit excellent catalytic activity and selectivity toward the desired hydrocarbons. The presence of sulfur is not considered as good as for the FTs process because the derivate of sulfur creates choking during the reaction in a fixed bed reactor. MOFs prepared by ferric sulfate exhibit the highest amount of 52.50% light hydrocarbons. The calcined ferric sulfate (P.MOF.S) dramatically impacts the carbon chain distribution and suppresses the formation of light hydrocarbons. The calcined ferric nitrate (P.MOF.N) attributes the highest 93.27% diesel production for Fischer Tropsch synthesis. The total amount of Oxygenates was observed as low in carbon chain distribution, and this low amount is considered an excellent advantage for hydrocarbon production. Therefore, this study concludes that the calcined MOF prepared by ferric nitrate exhibit superb selectivity for liquid hydrocarbons.

## Data Availability

The original contributions presented in the study are included in the article/supplementary material, further inquiries can be directed to the corresponding authors.
